# Investigation of human defence responses in a virtual reality environment: an EEG study

**DOI:** 10.3389/fnhum.2026.1924058

**Published:** 2026-09-30

**Authors:** Vidisha Maithani, Manish Kumar Asthana

**Affiliations:** 1 Department of Humanities and Social Sciences, Indian Institute of Technology Roorkee, Roorkee, India; 2 Department of Design, Indian Institute of Technology Roorkee, Roorkee, India

**Keywords:** defence response, fight-or-flight, research domain criteria, threat imminence continuum, virtual reality

## Abstract

A predatory imminence continuum displays the perceived imminence of threat to visualise the risk to the prey. Most human studies have utilised static paradigms that may lack ecological validity and limit the exploration of defensive behaviours. Therefore, the present study investigates Fight/ Flight defence responses and underlying neural correlates using EEG under varying conditions of threat proximity and escape availability using a virtual reality (VR) environment. The experiment consisted of a desktop VR environment, including training and experimental phases (5 conditions). Conditions 1 to 4, except 5, included a threat avatar. The conditions differed based on threat proximity (distal/ proximal) and participant’s escapability (escape/no-escape). Twenty trials were presented, with each condition presented 4 times. Participants responded with a Fight/Flight response to the threat, followed by a 10-s rest scene and a 5-s fixation. A significant difference in reaction time was observed, indicating distinction based on threat proximity and escapability. A heightened Fight response was observed across all conditions. This pattern may reflect the absence of consequences associated with defensive actions compared to real-life scenarios. For the electrophysiological analysis, ERP analysis indicated a significant difference in the Late frontal activity at the F1 electrode across all conditions. This may suggest tentative cognitive processing during the period surrounding defensive response (Fight or Flight) selection and execution. Together, the findings indicate that although the lack of consequences may have promoted the Fight response more in the VR scenarios, the neural responses may have shown differences in cognitive processing.

## 1 Introduction

One of the significant roles of fear in an organism’s lifespan is to defend it from threats. These defensive behaviours may deter predators and increase the prey’s chances of survival. It integrates fear as an essential part of the survival mechanism (Cannon, 1929; Bolles, 1970). The stage of defence a prey is currently on is determined by its perception of the current level of risk (Fanselow and Lester, 1988). Fanselow and Lester (1988) created a predatory imminence continuum (PIC) that depicts the psychological perception of threat imminence, thereby visualising the current level of risk for the prey. The continuum ranges from low to high, with one end denoting complete safety and the other depicting the chances of being consumed by a predator. The imminence is not dependent solely on physical distance, time, or probability, but also on the psychological distance from the predator (Fanselow, 2022; Darves-Bornoz et al., 2008; May and Wisco, 2016). The continuum is broadly divided into the pre-encounter phase, the post-encounter phase and the circa-strike phase (Fanselow, 2022; Schmidt et al., 2015). The pre-encounter phase is the first or initial level in the predatory imminence continuum. This phase occurs when an organism ventures from a safe position to a potentially threatening environment. The term pre-encounter refers to a situation in which an organism has not yet detected any actual threat in the environment, such as when an animal leaves its shelter to forage in a potentially risky environment (Fanselow, 2022; Smith et al., 2022; Mobbs et al., 2020). In the post-encounter phase, a predator has been detected, and the prey is unsure whether a physical attack is imminent. The behaviours involved include avoiding detection by the predator, i.e., the flight response, and, if detected, reducing the probability of attack (Fanselow, 2022). When the predator is in immediate surrounding contact with the prey, imminence increases, and the behaviour elicited by the prey is vigorous and active. The behaviour elicited during this phase is the circa-strike or the fight response, as the escape routes are unavailable and the threat is in closer proximity to the prey (Fanselow and Lester, 1988; Stefan Bracha et al., 2004). This indicates that threat proximity influences a prey’s assessment of its current position on the predatory imminence continuum (Ellard and Eller, 2009; Ydenberg and Dill, 1986; Ellard and Goodale, 1988).

Various studies have replicated the threat imminence continuum in rodents, and some studies on threat scenarios have replicated findings in human participants (Blanchard et al., 2003). Human defence responses (fight-or-flight) are evolutionarily significant behaviours that influence our survival, threat perception, and decision-making under threatening situations (Roelofs, 2017). However, investigating these responses in real-world scenarios presents ethical, methodological and practical challenges. In humans, threat scenarios have been utilised to measure approach or avoidance responses to threat (Åhs et al., 2015; Caroline Blanchard et al., 2001). The scenarios varied based on the threat’s magnitude, the distance between the threat and the participant, the participant’s ability to escape, and the presence of a hiding place (Blanchard et al., 2011). One limitation of such studies is that text-based threat scenarios may not elicit the same levels of presence, arousal, ecological validity, and dynamism as real-life threatening scenarios (Parsons, 2015; Freeman, 2007). Virtual reality (VR) provides the platform for a controlled yet dynamic environment that allows systematic investigation of defence responses under simulated threat conditions. Understanding these responses may, thus, contribute to a foundational understanding of human defence responses and to the future expansion of the threat imminence continuum. Studies on VR scenarios that address the threat imminence continuum have mostly focused on avoidance and escape responses to an AI predator (Brandão et al., 2022; Lee et al., 2022). According to the current published literature, the fight response has been the least explored defence response in the threat imminence continuum among human participants. The current objective thus proposes to investigate the fight-or-flight response in human participants. The study hypothesises that threat conditions will elicit observable fight-or-flight defensive responses and that differences will emerge based on threat proximity and the potential for escape. To evaluate the effectiveness and experiential validity of the VR paradigm and environment, subjective VR-based questionnaires were incorporated. Azher et al. (2023), compared screen-based and headset-based versions for the virtual reality simulation and reported comparable performance, usability, cognitive load, and emotional experience using the two modalities. Thus, screen-based virtual reality simulations provide an accessible alternative to Head Mounted Display (HMD) VR simulation, and is also utilised in our study. Previous research associated the Late Positive Potential (LPP) with action preparation and motivational engagement (Lu et al., 2023). Since responding to threats requires the selection and preparation of an appropriate behavioural response to an imminent threat, we hypothesised that the frontal LPP activity will be modulated during the period surrounding defensive response (Fight or Flight) selection and execution. Accordingly, the current study examined frontal LPP activity in response-locked epochs, with the onset of the defence responses as the temporal reference point.

The National Institute of Mental Health (USA) developed a framework known as the Research Domain Criteria (RDoC) to develop new ways of categorising disorders based on dimensions of observable brain functions and disorders (Definitions of the RDOC Domains and Constructs, n.d.). From the criteria, Negative Valence Systems include responses to aversive events, such as fear, anxiety, and loss. Three constructs within the negative valence system are Potential Threat, Acute Threat, and Sustained Threat (Fanselow, 2022). Defensive threat responses are observed under these three threat conditions. The current study focuses on Potential and Acute threats.

## 2 Methods

### 2.1 Participants

Two sets of participants were recruited separately for the preliminary study and the main experiment. Eleven participants (mean age = 24.72, SD = 2.76), aged 18 to 30 years, were selected for this preliminary study. *A priori* power analysis through G*Power 3.1 software (Faul et al., 2007) was conducted to determine the sample size. Assuming an effect size of 0.25 and a power of 0.80, for the repeated measures ANOVA within factors, a minimum sample size of 24 was required. For the main experiment, 39 participants (mean age = 22.95, SD = 3.73) (Males = 31; Females = 8) from the same age range participated from the research institute. For the behavioural analysis, 39 participants were included. For the EEG analysis, data from 29 participants were included, as the excluded data had technical issues, excessive noise and artifacts that rendered the data unsuitable for analysis. For inclusion criteria, we selected participants with normal or corrected-to-normal visual acuity (verbal self-report), normal auditory acuity (verbal self-report and no hearing aids), and familiarity with English. The exclusion criteria were participants with colour blindness, a medical history of neurological or diagnosed psychiatric disorders, pregnancy, or any other medical conditions. Participants who were under the influence of alcohol or any other psychoactive substances were also excluded from the study. Informed consent was obtained from each participant at the beginning of the experiment, and all the procedures were thoroughly explained. The procedures adhered to the Institutional Human Ethics Committee’s code of ethics and conduct. The study received ethical approval from the Institutional Human Ethics Committee (Letter No. IITR/IIC/24/14).

### 2.2 Preliminary study

A preliminary study was conducted to select an auditory threat stimulus for the threat avatar, assess the participant avatar’s walking speed, standardise the perception of the threat as distal or proximal to the participant across conditions, and standardise the environment. Participants were presented with 5 auditory stimuli (Freesound, n.d.) via Psychopy software (v2025; Peirce et al., 2019). (Individual audio files were used in accordance with their respective Creative Commons licenses, and required attributions are provided in the Supplementary file). Each audio was rated on the Self-Assessment Manikin (SAM) Likert rating scale on valence (1 = Unpleasant to 9 = Pleasant) and arousal (1 = Calm to 9 = Excited) dimensions (Bradley and Lang, 1994). Based on the lowest mean valence (Mean = 2.09, SD = 1.04) and the second-highest mean arousal (Mean = 5.82, SD = 2.04), Audio 5 was selected for the threatening avatar. To standardise perceived distance and walking speed, the VR scenario was presented to participants as it would appear in the main experiment. Participants were presented with a familiarisation scene and a no-threat condition scene, during which they assessed the walking speed of the first-person participant avatar while exploring the VR environment. The familiarisation scene allowed them to become accustomed to the keyboard and mouse controls and to navigate the environment before assessing their avatar’s movement speed. After the familiarisation and no-threat scenes, participants rated the walking speed on a 5-point Likert rating scale (1 = Too slow, 2 = Slow, 3 = Just right, 4 = Fast, 5 = Too fast). Ratings were converted to numerical values for calculation. The selected speed of the avatar was rated approximately slow in the familiarisation scene (Mean = 2.27, SD = 0.79) and approximately Just right in the no-threat (NT) scene (Mean = 2.73, SD = 0.47), indicating that they perceived the movement speed as appropriate after familiarisation with the environment. The walking speed was adapted accordingly. To assess the perceived distance of the threat avatar across different conditions, participants were presented with 4 threat-based conditions (Distal Threat Escape: DTE, Distal Threat No Escape: DTNE, Proximal Threat Escape: PTE, and Proximal Threat No Escape: PTNE). For each condition, participants rated the avatar’s proximity on a Likert scale (Distal = 1, Not Sure = 2, Proximal = 3). Participants perceived the distance of the Threat avatar as Distal, for the Distal Threat Escape (Mean = 1.36, SD = 0.81) and Distal Threat No Escape (Mean = 1.36, SD = 0.81). For the Proximal Threat Escape (Mean = 3, SD = 0.00) and Proximal Threat No Escape (Mean = 2.55, SD = 0.82), participants perceived the threat avatar as Proximal (for more details, refer to Supplementary Figures S1–S4).

### 2.3 Materials

#### 2.3.1 Pre-experiment questionnaires

The questionnaires at the beginning of the experiment were administered using the PsychoPy software (v2025; Peirce et al., 2019). The State–Trait Anxiety Inventory (STAI-trait) questionnaire (Spielberger et al., 1983) was administered to assess participants’ trait anxiety. The Positive and Negative Affect Schedule (PANAS) questionnaire (Watson et al., 1988) was administered to measure participants’ positive and negative affect. In addition, the Santa Barbara Sense-of-Direction scale (Hegarty, 2002) was administered to assess participants’ sense of direction, as the experiment involved navigating a virtual environment (for more details, refer to the Pre-Experiment Questionnaire section in the Supplementary file).

#### 2.3.2 Post-experiment virtual reality questionnaires

A set of Virtual Reality (VR) questionnaires was administered post-experiment using PsychoPy software to assess participants’ overall experience in the VR environment. These include a 5-item Body-illusion questionnaire (Penaud et al., 2023) to assess agency, self-identification, self-location and control in the environment rated on a 7-point Likert scale (−3 = Strongly Disagree, 0 = Neutral, 3 = Strongly Agree). A 9-item (modified to include only relevant items) i-group presence questionnaire (IPQ) to assess the general presence, spatial presence, involvement, and experienced realism of the participants on a 7-point Likert scale (−3 to +3; The rating labels vary according to the question items) (Schubert et al., 2001). A 6-item user-experience questionnaire for VR locomotion on a 5-point Likert scale (0 = not at all, 1 = Slightly, 2 = Moderately, 3 = Fairly to 4 = extremely) was used to assess Immersion, Negative Affect, Tension, Challenge, Tiredness and Perceived Usability (Boletsis, 2020). A 5-item Subjective dread questionnaire was also administered to assess the level of dread in response to the VR locations in the environment and the threat avatar on a 10-point Likert scale (0 = no dread to 9 = maximum dread) and a 10-item virtual reality environment questionnaire on a 7-point Likert scale (1 = not at all to 7 = completely) was administered to assess the experience related to the threat avatar and the VR scenes (modified from Lu et al., 2023). For more details, refer to the Post-Experiment Questionnaire section in the Supplementary file.

#### 2.3.3 Virtual reality (VR) experimental design

A 3-dimensional virtual reality environment (screen-based) was designed using Unity (version 2022.3.14f1, Unity Technologies, US) to conduct the experiment. Throughout the experiment, the term “VR experiment” refers to the screen-based VR environment. The design consisted of an urban city area with 4 lanes, incorporating virtual objects obtained from the Unity Asset Store (Unity, 2000). A human threat avatar was also added to the scene as the threat stimulus, which animates with a running and punching animation when encountered by the participant. A box item was also included in the scene as the task stimulus for the participant. Auditory stimuli for the participant’s footsteps during movement, the threat avatar’s running, and the threat avatar’s punching attack were used to enhance the realism the VR environment. For the behavioural data recording, Unity was connected with MySQL server (Oracle Corporation, 2025) using php (The PHP Group, 2025).

#### 2.3.4 Electrophysiological data acquisition

An 8-channel electroencephalography (EEG) device (Mentalab GmbH, Germany) was used to collect electrophysiological data throughout the main experiment. The EEG device was connected to Unity through Lab Streaming Layer (LSL) (Kothe et al., 2025) and it was also used to record the electrophysiological data. The electrodes were placed at the Frontal sites (Fp1, Fpz, Fp2, F3, F1, Fz, F2, and F4) using the international 10–10 system. The selected electrode sites were chosen based on their relevance implicated in social threat processing (Lu et al., 2023), affective processing (Abado et al., 2024), and attentional engagement towards threatening stimuli (Holmes et al., 2003; Bar-Haim et al., 2007), during emotionally salient tasks. Enhanced Late-positive Potential (LPP) amplitudes are observed over fronto-central regions during action preparation and motivational engagement (Lu et al., 2023). Given that defensive responses involve threat evaluation and preparation for adaptive behavioural actions, we hypothesised that a significantly enhanced LPP amplitude may be observed over frontal sites during the fight-or-flight response.

### 2.4 Procedure

#### 2.4.1 Experiment

At the beginning of the experiment, participants were presented with the STAI, PANAS, and Sense of Direction questionnaires using PsychoPy software. The main experiment was presented in the Unity software platform. Participants were presented with a 3D virtual reality (VR) environment from a first-person perspective (1PP) on a desktop screen. The VR scenario consisted of an urban city view. The participants’ avatar started from the ‘Start’ sign in the scenario. Their avatar could move and explore different lanes (Lanes 1, 2, 3, and 4). The main experiment consisted of 2 phases: the Training and Experimental phases. The training phase consisted of 5 trials. In the training phase, participants were instructed to explore the environment and locate and move towards a Box stimulus that was presented pseudo-randomly in one of the lanes. After locating the Box, the participants had to move towards the ‘Stop’ sign at the end of the road, which marked the trial’s end. The participants familiarised themselves with the 3D environment and the assigned keys for the avatar’s movement. After the 5th trial, the participants were shown a prompt on the screen asking if they wanted to move to the main experiment or needed more practice. If participants selected further practice, they were shown one additional trial before the main experiment. The inclusion of training phase was intended to allow participants to reach a stable level of task performance and familiarity with the VR environment before the Experimental phase trials. A study by Chen et al. (2020) reported that participants may require practice trials to achieve performance stability in addition to experiment trials. The Experimental phase consisted of 20 trials. As shown in Figure 1, it consisted of 5 conditions: Distal Threat Escape (DTE), Distal Threat No Escape (DTNE), Proximal Threat Escape (PTE), Proximal Threat No Escape (PTNE) and No Threat (NT). The Distal and Proximal Threat conditions consisted of threats at Distal and Proximal distances, whereas the Escape and No escape conditions referred to the location of the participants’ avatar in the scene. If the participant’s avatar is at the closed end of the lane with no area to escape when the threat appears, it is a No Escape condition. If the participants’ avatar is at the open end of the lane with an area to escape, it is an Escape condition. No Threat condition consisted of no threat avatar. Each condition was presented 4 times, and pseudo-randomised across the experiment. The number of trials selected (4 trials for each condition) provided repeated observations of each condition while limiting excessive repetition and potential habituation. The number of trials are consistent with previous work examining performance stability in VR environment tasks, which observed that 3–6 trials suffice to achieve performance stability (Chen et al., 2020). The presentation of the box across the 4 lanes was also pseudo-randomised to minimise any association between a particular lane and threat. In the Experimental phase, participants were instructed that the main task of locating a box in the lanes would remain the same as in the training phase. However, during exploration, they may encounter a Threat avatar that approaches them. The task then switched to having participants select the Fight (Q key) or Flight (P key) response. They were given 7 s to respond from the onset of the threat-avatar stimulus, after which the trial ended (Cheng et al., 2018). Once a response was chosen or the 7 s elapsed, the trial ended, whichever occurred first. Failure to respond during the provided time window resulted in no response. A 10-s rest period was presented after each trial, followed by a 5-s fixation. After the Experimental phase, the participants were presented with the Post-Experiment VR Questionnaire in PsychoPy.

**Figure 1:**
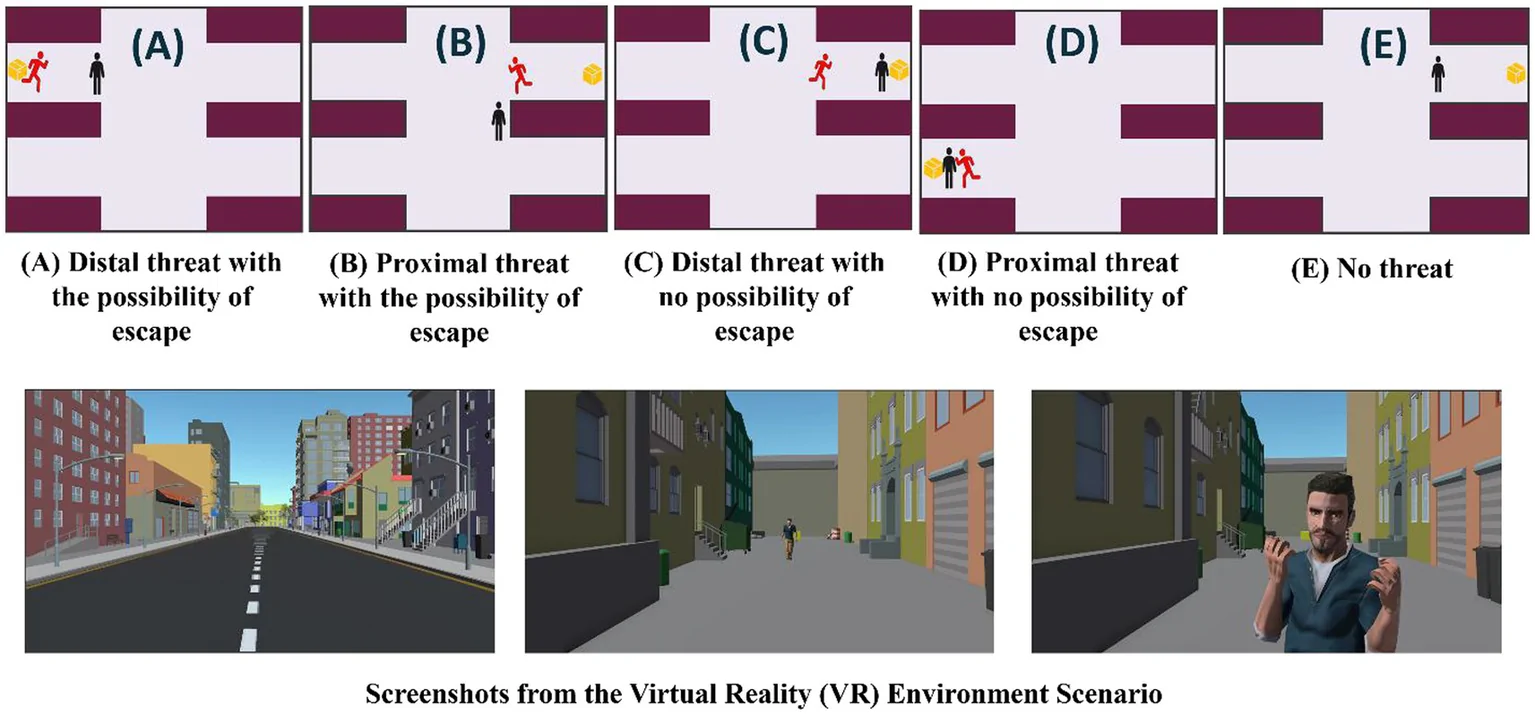
Schematic representation of the 5 conditions and screenshots from the VR environment. In the top row, the black icon represents the participant’s avatar, the red icon represents the threat avatar, and the box icon represents the box stimulus in the VR environment. **(A)** Distal threat with the possibility of escape, **(B)** Proximal threat with the possibility of escape, **(C)** Distal threat with no possibility of escape, **(D)** Proximal threat with no possibility of escape, and **(E)** No threat. The screenshots of the VR environment represent urban city environment, distal threat and proximal threat respectively.

## 3 Data analysis

### 3.1 Behavioural analysis

Behavioural responses were analysed to assess participants’ defence responses (Fight or Flight) and reaction times across the 4 threat-based conditions (DTE, DTNE, PTE, and PTNE). The No Threat (NT) condition did not include the threat avatar. The end of the trial was marked by the completion of the task (finding the box stimulus and reaching the Stop sign). Since it did not include a threat stimulus, no defence response was recorded for the No Threat condition and thus, was not included in the behavioural responses and reaction time analyses. Behavioural responses were analysed at the trial level. Since Fight and Flight responses constituted mutually exclusive binary response outcomes, trial-level generalised linear mixed-effects models (GLMM) with a binomial distribution and logit link was assessed. Participants were included as a random intercept, while conditions (DTE, DTNE, PTE, and PTNE) were included as a fixed effect. Two GLMMs were conducted. First, for trials with defensive responses, the effect of Condition on Fight and Flight selection was examined. No-response trials were excluded from analysis. Second, response occurrence was examined, including all trials and testing whether Condition affected by the probability of making a response versus not making a response.

Reaction times for the defence responses were recorded based on the amount of time the participant took to respond within the 7-s time window following the appearance of the threat avatar stimulus in the scene. A repeated-measures ANOVA was conducted to examine the difference in reaction time across the 4 conditions.

Reaction time (RT) were also analysed at the trial level using a linear mixed-effects model. Trials with no response (no Fight or Flight) were excluded from the RT analysis. As the residual of the model fitted to raw RTs presented substantial positive skew, RTs were log-transformed prior to the final analysis. Conditions (DTE, DTNE, PTE, and PTNE), Response (Fight and Flight), and Condition x Response were included as fixed effects. Participants were included as a random intercept. The model was estimated using restricted maximum likelihood (REML), with Satterthwaite approximation for the denominator degrees of freedom.

### 3.2 Electrophysiological analysis

#### 3.2.1 Preprocessing

EEG data were collected using the international 10–10 system montage, with 8-channel electrodes throughout the experiment. The data were preprocessed and analysed using the MNE Python library. A DC offset correction was applied to the data to remove slow baseline drifts and any voltage shifts unrelated to neural activity. A bandpass filter from 0.1 to 30 Hz was applied to remove slow drifts and high-frequency noise (Lu et al., 2023). A notch filter at 50 Hz was applied to attenuate any remnant line noise. An average reference was used during recording. Impedance for the recording was maintained below 10 kOhm. Independent Component Analysis (ICA) was used to correct any ocular and movement artifacts. The data was further visually inspected to remove any remaining artifacts and bad segments, thereby maintaining data quality. EEG data from the 29 participants retained for ERP analysis yielded a maximum of 580 trials. From 580 trials, after EEG preprocessing and artifact rejection, 494 trials (85.2%) were retained for ERP analysis. Trial retention was 82.8% for DTE (96 of 116 trials), 83.6% for DTNE (97 or 116 trials), 82.8% for PTE (96 of 116 trials), 80.2% for PTNE (93 of 116 trials), and 96.6% for NT (112 of 116 trials).

#### 3.2.2 Event-related potential analyses

Following preprocessing, the data were segmented into 12.2 s time window around the end of the trial for each condition. For threat-based conditions (DTE, DTNE, PTE, and PTNE) the end of the trial was marked by the Fight or Flight response. These epochs were response-locked to assess electrophysiological activity associated with the defence response selection and execution. For the No threat (NT) condition, the end of the trial did not include the Fight or Flight response, but only the end of the trial. The NT condition trials were aligned to trial termination rather than a behavioural response. A time window from −7.2 s before the onset and 5 s after the onset was extracted and epoched from each trial for each participant. A baseline correction of 200 msecs (−7.2 to −7 s) was applied to each epoch. The baseline duration was selected as −7.2 to −7 s, based on the temporal constraints of the experimental paradigm. During this time the threat was not present in the scene and the participant’s avatar was exploring it. Since epochs were response-locked, the temporal relationship between the baseline and subsequent threat onset varied according to the condition. However, since there was 7 s to respond after the threat onset, we know that 200 msecs before 7 s (−7.2 to −7 s) interval relative to the response necessarily preceded threat onset, including on trials with the maximum permitted response latency. The segmented epochs were averaged for each condition (DTE, DTNE, PTE, PTNE, and NT). Epochs were averaged for each condition and participant. Of the total 580 epochs, 494 epochs (85.2%) were retained for ERP analysis. A grand average was calculated for all the participants for each condition. Repeated-measures ANOVA were conducted for Frontal electrodes (Fp1, Fpz, Fp2, F3, F1, Fz, F2, and F4) to assess the mean ERP amplitudes for Late Positive Potentials (LPP) within the 500–1,000 msecs time window across the 5 conditions (DTE, DTNE, PTE, PTNE, and NT) to assess any decision-making and threat-based emotional stimuli neural responses around the period of defensive response selection and execution. An exploratory repeated-measures ANOVA for single electrodes was also conducted to assess any late frontal activity within the 1,500–2000 msecs time window for all the conditions.

## 4 Results

### 4.1 Pre-experiment questionnaires

#### 4.1.1 STAI-Y (Trait) and PANAS

STAI-Trait anxiety and PANAS scores questionnaires were administered to assess the pre-experiment baseline anxiety and affect levels. The STAI-Y (Trait) scale scores showed medium anxiety (Mean = 42.21, SD = 10.05) (Joshi et al., 2023) and PANAS questionnaire (Positive Affect: Mean = 35.77, SD = 6.87; Negative Affect: Mean = 17.69, SD = 6.61) (Watson et al., 1988) scores were within the normative range, suggesting that any examined effects are unlikely due to pre-experimental emotional states.

#### 4.1.2 Santa Barbara Sense of Direction Scale

Santa Barbara Sense of Direction Scale (SBSOD) scores were analysed to assess participants’ self-reported navigational and spatial orientation ability prior to the experiment. The scores indicated that participants started with a moderate level of perceived sense of direction (Mean = 4.57, SD = 1.08) (Hegarty, 2002).

### 4.2 Post-experiment questionnaires

#### 4.2.1 Body-illusion questionnaire

Responses to the questionnaire indicate a neutral level of Agency (Mean = 0.69, SD = 1.70), Self-identification (Mean = 0.28, SD = 1.88) and Self-location (Mean = 0.26, SD = 1.60) suggesting that participants felt a neutral sense of control over their actions and embodiment in the virtual environment. Control ratings (Mean = −1.15, SD = 1.29) indicate that participants felt slightly more control over their bodies than neutral.

#### 4.2.2 I-group presence questionnaire

Responses to the questionnaire indicate a neutral to moderate level of General presence (Mean = 0.56, SD = 1.88) Spatial presence (Mean = 0.28, SD = 1.17), Involvement (Mean = 0.56, SD = 1.85) and Experienced realism (Mean = 0.08, SD = 0.94) in the virtual environment.

#### 4.2.3 User experience questionnaire

For the user experience, responses to the questionnaire indicate a fairly high level of immersion (Mean = 3.10, SD = 0.85). A slight negative effect (Mean = 1.74, SD = 1.23) and tension (Mean = 1.03, SD = 1.23). The participants found navigating and understanding the environment very less or not at all challenging (Mean = 0.44, SD = 1.05). They found slight Tiredness (Mean = 1.05, SD = 1.26) and fairly high perceived usability (Mean = 3.33, SD = 0.98).

#### 4.2.4 Subjective dread ratings

Subjective dread ratings for the virtual reality scenes from the environment, as expected, are highest for the proximal threat (Mean = 7.69, SD = 2.05), followed by distal threat (Mean = 6.77, SD = 2.16), pre-encounter (Mean = 3.18, SD = 2.48) and safety (Mean = 2.21, SD = 2.25) scenes in decreasing order of perceived dread. Subjective dread ratings increased as the threat became more imminent, thus indicating that the threat manipulation successfully increased perceived threat intensity in the environment.

#### 4.2.5 Virtual reality environment questionnaire

Ratings on the questionnaire for the environment and the stimuli in the environment indicate that participants perceived neutral level of realism (Mean = 4.62, SD = 1.46), violence (Mean = 4.36, SD = 1.77), and assault (Mean = 4.59, SD = 1.65). They perceived a slight higher than neutral level of fear of threat avatar (Mean = 5.13, SD = 1.47), fear of approaching avatar (Mean = 6.03, SD = 1.48) and vulnerability (Mean = 5.05, SD = 2.11). They also perceived higher fear for proximal threat (Mean = 6.33, SD = 1.30) as compared to distal threat (Mean = 4.28, SD = 1.64). Ratings for the possibility of escape (Mean = 3.54, SD = 1.55) and no escape (Mean = 3.82, SD = 1.83) were comparably neutral (For more details, refer to Supplementary Table S1).

### 4.3 Behavioural results

#### 4.3.1 Defense responses (Fight and Flight responses)

Across the 624 trials, defensive responses were made on 609 trials (97.6%), including 424 Fight responses (69.6% of responses) and 185 Flight responses (30.4% of responses) and No response occurred on 15 trials (2.4% of all trials). A trial-level generalised linear mixed-effects model (GLMM) analysis was conducted to assess whether the threat Conditions influenced occurrence of the behavioural responses (response versus no-response). Participant was included as a random intercept and Condition as a fixed effect. The analysis revealed no significant effect of Condition on the occurrence of response, *F*(3,620) = 0.18, *p* = 0.91. A second trial-level GLMM examined whether Condition had an influence on Fight vs. Flight response. Participants were included as a random intercept. There was no significant effect of Condition on Fight vs. Flight selection, *F*(3,605) = 0.64, *p* = 0.59. Although overall Fight responses were descriptively more frequent than Flight responses, the relative distribution of Fight and Flight did not significantly differ across the threat Conditions (DTE, DTNE, PTE, and PTNE) (Figure 2).

**Figure 2:**
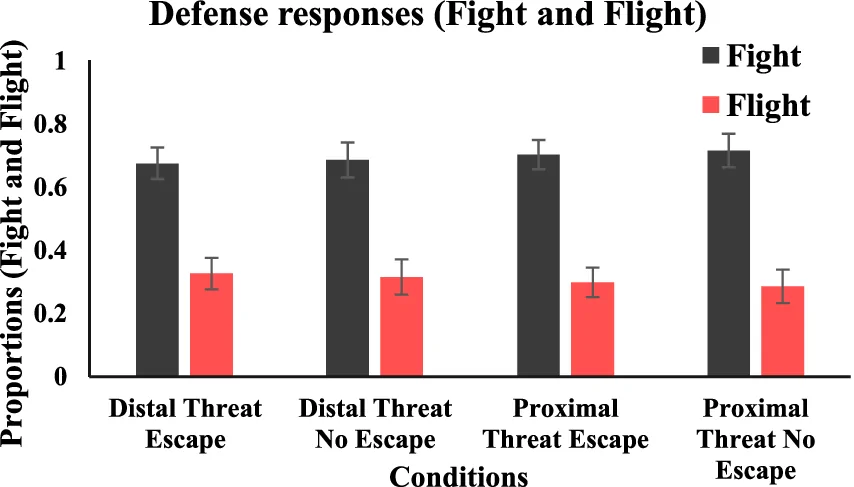
Graph depicting mean of defence responses (Fight or Flight) proportions across four threat conditions. Proportions were calculated among trials in which a defensive response occurred; no response trials were excluded.

#### 4.3.2 Reaction time

A repeated-measures ANOVA was performed to compare Reaction Time (RT) across the 4 conditions (DTE, DTNE, PTE, and PTNE). As shown in Figure 3, there was a significant difference in reaction times among the four conditions, *F*(3,114) = 4.63, *p* < 0.01, ηp^2^ = 0.11. Pairwise comparisons with Bonferroni correction revealed that the reaction time of the Distal Threat Escape (Mean = 2.05, SD = 0.84) was greater than the Proximal Threat No Escape condition (Mean = 1.58, SD = 0.74), *p* < 0.01.

**Figure 3:**
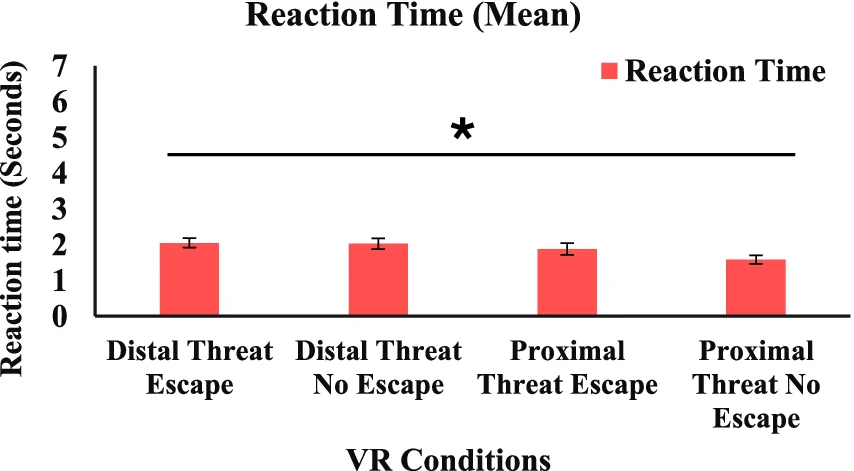
Graph depicting mean of reaction time of defence responses across four threat conditions.

Trial level linear mixed-effects analysis revealed a significant main effect of Condition on log-transformed RT, *F*(3,564.11) = 9.87, *p* < 0.01. Bonferroni adjusted pairwise comparisons showed a significant difference between DTE and PTE (Mean Diff = 0.188, SE = 0.06), *p* < 0.01 [95%CI (0.04, 0.34)], between DTE and PTNE (Mean Diff = 0.29, SE = 0.06), *p* < 0.01[95%CI, (0.14, 0.44)] and DTNE and PTNE (Mean Diff = 0.21, SE = 0.06), *p* < 0.01, [95% CI, (0.06, 0.37)]. No significant differences were observed between DTE and DTNE (Mean Diff = 0.08, SE = 0.06), *p* = 0.98, [95%CI, (−0.07, 0.23)], DTNE and PTE (Mean Diff = 0.11, SE = 0.06), *p* = 0.34 [95%CI, (−0.04, 0.26)], and PTE and PTNE (Mean Diff = 0.10, SE = 0.06), *p* = 0.46, [95%CI, (−0.05, 0.26)]. There was also no significant main effect of Response, *F*(1,596.52) = 0.04, *p* = 0.84, and interaction effect of Condition x Response *F*(3,569.88) = 1.82, *p* = 0.14.

### 4.4 Electrophysiological results

Repeated-measures ANOVA were conducted for Frontal electrodes (Fp1, Fpz, Fp2, F3, F1, Fz, F2 and F4) to examine the differences in Late-Positive Potentials (LPP) within the 500–1,000 milliseconds time window after the end of the trial onset in all conditions. As shown in Figure 4, the analysis did not reveal a significant difference across conditions [*F*(1.84, 51.52) = 0.65, *p* = 0.52, ηp^2^ = 0.02].

**Figure 4:**
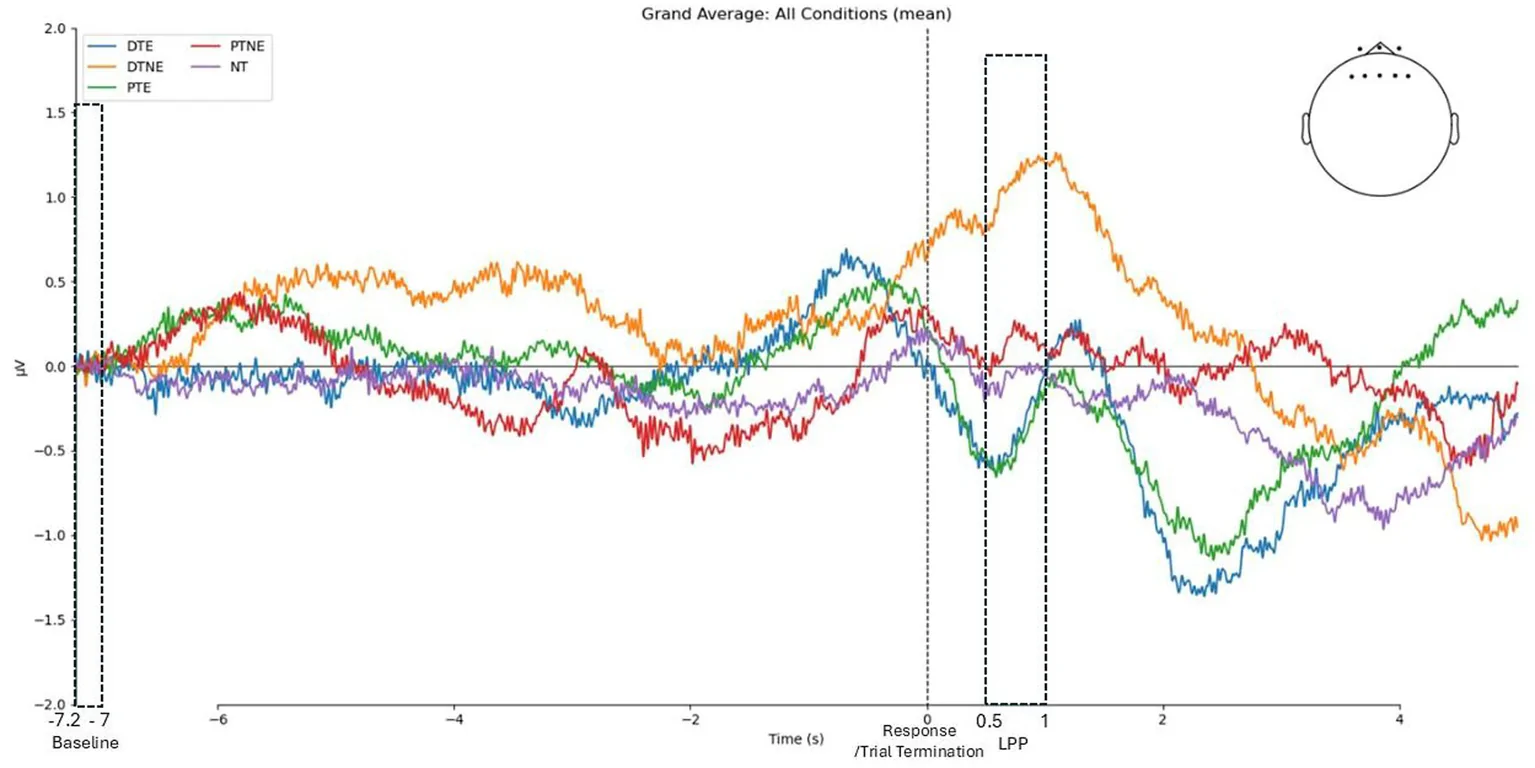
Grand average ERP graph for all conditions and all channels.

As shown in Figure 5, later exploratory analyses of single electrodes revealed a significant difference at electrode F1 within the 1,500–2000 msecs time window, indicating probable late frontal activity across the 5 conditions (DTE, DTNE, PTE, PTNE and NT), *F*(4,112) = 3.11, *p* = 0.02, ηp^2^ = 0.10. No significant differences were observed on the remaining electrodes. (For more details, refer to Supplementary Figures S5–S11). This F1 effect originates from post-hoc exploratory testing without correction for multiple comparisons; accordingly, it is purely descriptive and cannot be treated as confirmatory statistical evidence.

**Figure 5:**
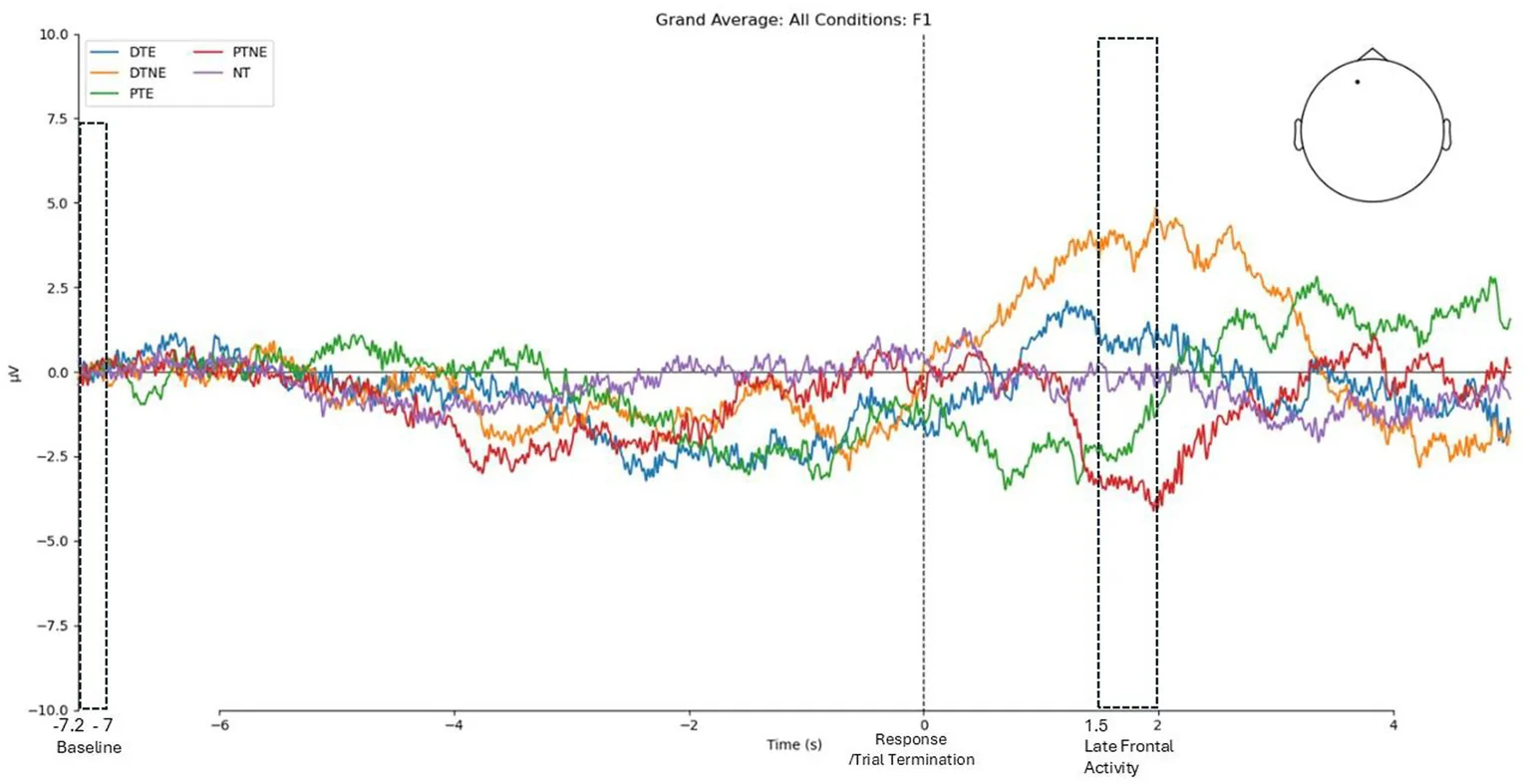
Grand average ERP graph for all conditions, F1 electrode.

## 5 Discussion

The behavioural results of our study indicate that there was no significant effect of threat-based conditions (DTE, DTNE, PTE, and PTNE) on the Fight vs. Flight defence responses. This suggests that the differences in threat proximity and the possibility of escape did not bias participants’ defence responses toward selecting one defence strategy over another. Similarly, examining response occurrence revealed no significant effect of threat-based conditions on whether participants chose a response or not. Thus, the manipulation of threat proximity and participant’s escapability did not significantly alter the selection of Fight or Flight responses or the likelihood of making a response. This contrasts with findings from animal research based on the threat imminence continuum, which suggest that animals engage in circa-strike (fight) responses under proximal threat with no-escape conditions, and exhibit flight defence responses under distal threat and with the possibility of escape conditions (Fanselow and Lester, 1988). Despite the absence of differences in defence responses across conditions, a consistent pattern emerged across all the 4 conditions where the fight defence response occurred significantly more frequently, approximately twice as frequently as the flight defence response. The higher frequency of fight responses compared with flight responses may be attributable to the design of the experimental task. According to the literature, the threat imminence continuum focuses primarily on the psychological distance of the threat (distal vs. proximal) and the organism’s escapability (Fanselow, 1994). These factors were incorporated in the present experimental design. However, once the defence response was chosen (Fight or Flight), the trial ended for the particular condition. In natural contexts, defence responses are often followed by consequences. For example, a fight defence response may result in successfully neutralising the threat or sustaining physical injury. Similarly, a flight defence response may result in either the organism’s successful escape or its capture by the threat. Since the current experiment did not include any consequences for the chosen defence responses, it may have reduced the perceived survival cost–benefit associated with defence responses in natural contexts; thus, the participants may have inclined towards the selection of the fight response. This differs from defensive responses in animals-based predatory imminence continuum paradigms, where the consequences of the defensive behaviour are directly relevant to survival and forms part of the organism’s cost–benefit evaluation of the available responses. This observed pattern suggests that, in addition to threat proximity and escapability, the anticipated consequences of defence responses may also contribute to the selection of defence responses. However, as this possibility was not directly tested in the current study, it should be considered speculative. Other factors that may have contributed are individual response preference, gaming experience, and perceived agency in the virtual environment. An additional observation reveals that the escapability manipulation may have been less effective than the threat proximity manipulation. Subjective ratings in the post-experiment Virtual reality environment questionnaire suggested limited differentiation between the Escape and No-Escape conditions, potentially suggesting that participants may not have perceived these conditions as strongly distinct in terms of perceived escape possibility. This could also indicate a possible weaker behavioural impact of the escapability manipulation and may partly explain absence of condition-specific differences in Fight and Flight responses. Therefore, the behavioural differences observed among the conditions cannot be interpreted as unequivocal evidence of the effects of perceived escapability. The absence of behavioural differences between these conditions should be interpreted within the context of the strength of the escapability manipulation.

In contrast to the behavioural responses, the reaction times of the behavioural responses differed significantly across conditions (DTE, DTNE, PTE and PTNE). The log-transformed reaction times suggested that the reaction times of the DTE condition were greater than those of both the PTE and PTNE, as well significantly longer reaction times in DTNE than PTNE condition. This aligns with the expectations of the threat evaluation process. When the threat is distal with a possibility of escape, participants have more time to assess the situation and determine an appropriate response. However, when the threat is proximal with no escape, the immediacy of the threat requires a faster response (Mobbs et al., 2020; Fanselow, 1994). This finding is consistent with the notion that an increase in threat imminence decreases the available deliberation time, leading to faster defence responses. The reaction time findings thus suggest that the threat manipulation influenced the timing of the defence responses, even though it did not significantly effect which defence response was selected. Taken together, these paradigm-specific considerations suggest that the behavioural findings should be interpreted with the specific characteristics of the current experimental design. The limited subjective differentiation between the escapability conditions constrains the conclusions that can be derived from the role of perceived escapability, whereas the absence of consequences following defence responses limit direct comparison with defensive behaviour in naturalistic and animal-based predatory imminence continuum paradigms. In addition, the gender imbalance of the sample limits generalisability of the experiment. Thus, the present findings provide evidence regarding selection of defensive responses within a controlled desktop-based virtual threat environment but should not be interpreted as demonstrating the threat proximity and participants’ escapability necessarily produce the same response patterns as those observed under strongly differentiated escape conditions, consequential outcome, or more demographically diverse populations.

In addition to the behavioural responses, the electrophysiological (EEG) recording was examined to assess the neural processes associated with threat perception and defensive responses in different conditions (DTE, DTNE, PTE, PTNE and NT) around the period of defensive response selection and execution. The ERP analysis focused on the Late Positive Potential (LPP) that is associated with task-relevant and emotional stimuli (Gable et al., 2015; Lu et al., 2023). No significant differences were observed in LPP across conditions when activity was averaged across the frontal electrodes cluster. However, during later analysis of single electrodes, a significant difference indicating a localised effect was observed for the F1 electrode, reflecting late frontal activity across conditions. In our study, stimulus onset was marked at the moment the trial ended, and the participant executed a defence response. Since the frontal regions are implicated in cognitive evaluation, the late frontal activity at F1 may tentatively reflect cognitive processing associated with the evaluation of the threat stimulus. However, this interpretation is speculative as the F1 effect was identified through post-hoc exploratory testing without correction for multiple comparisons and therefore cannot be considered confirmatory evidence. The absence of a significant LPP difference may relate to control over the threat. When an escape or another behavioural control is available, it may reduce the threat experience (Terburg et al., 2018). Also, the LPP amplitudes are larger under conditions of certainty (Johnen and Harrison, 2020). In our experiment, the position of the threat varied with proximity across conditions, and the threat may or may not be present (NT condition). This may have led to reduced non-significant LPP.

## 6 Limitations

Some limitations should be considered when interpreting the present findings. First, the small sample size may limit the statistical power of the experiment, along with an imbalance in male and female participant samples, which may limit the generalisability of the findings regarding potential gender-related differences in threat processing or defensive behaviour. The *a priori* G*Power analysis was conducted based on the behavioural repeated measures outcomes and was not specifically calculated for the ERP data analyses. In addition, the total number of trials included for the ERP analyses was reduced following filtering and artifact rejection. This may have reduced sensitivity to detect some effects, increasing the probability of both false-negative and false-positive findings, particularly for exploratory analyses. Therefore, the ERP results should be interpreted with caution and considered in the context of the above-mentioned limitations, rather than as definitive evidence of condition-related neural effects. Future studies with increased sample size and greater number of trials per condition, with a gender balanced sample, would provide a stronger statistical support for these effects and generalisability.

The current study assessed the threat imminence continuum among human participants and investigated their defence responses. Future studies can incorporate feedback or consequences for defence responses to gain a better understanding of these responses. Additionally, future studies may strengthen the escapability manipulation as it was less salient to participants than intended in the current study. Another limitation of the present study concerns the levels of immersion in the virtual environment. Since the design was presented on a desktop-based VR environment, the presence and embodiment reported by the participants were neutral-to-moderate. Future studies may employ head-mounted displays to increase the immersion and presence in the VR environment.

The post-experiment and pre-experiment questionnaires provided useful information regarding subjective experiences of the participants in the virtual environment. However, these measures were included to characterise the experimental experience and were not integrated in behavioural and electrophysiological analyses as covariates. The present study was designed primarily to examine the effect of experimental threat and proximity conditions on behavioural and electrophysiological outcomes. Given the sample size and the number of psychological variables considered, simultaneously including multiple subjective measures as covariates could reduce model stability and may complicate the interpretation of the experimental effects. Accordingly, these variables were not statically assessed in the present analyses. However, the possibility that subjective psychological states contributed to variability in the observed behavioural responses cannot be excluded. Future studies may assess and incorporate subjective experience as covariates related to defensive behaviour and neural findings.

In the context of electrophysiological measurement, EEG provides limited spatial resolution, thus it may restrict the precise localisation of the observed neural activity. Therefore, the localised effect observed on a single electrode may reflect measurement variability due to fluctuations in signal quality or noise, rather than a stable spatially distributed neural response. Thus, replication in future studies will be an important determinant to assess the localised effect reflecting a reliable neural pattern. Another methodological consideration of the response-locked ERP analysis concerns the use of a fixed baseline interval. Although the time window of −7.2 to −7 s was selected as a pre-threat onset period based on the 7 s maximum response window, the temporal distance between this baseline and threat onset varies across trials according to participants’ response latencies. Consequently, the baseline interpretation should be specifically within the temporal structure of the present experiment, rather than as a universally applicable baseline structure for response-locked ERP analyses. Future studies may further examine the robustness of the observed ERP findings using varied baseline strategies or stimulus-locked analyses.

## 7 Conclusion

The present study examined behavioural threat defence responses and electrophysiological correlates associated with different threat-based conditions in a virtual-reality environment. The behavioural findings indicated that participants chose the fight response more frequently than the flight response across conditions. The results suggest that defence responses are influenced not only by the proximity or imminence of the threat and the organism’s escapability, but also by the perceived consequences of defence responses. The reaction time, however, showed a significant difference across conditions, indicating that participants were able to distinguish among the different conditions. Electrophysiological analyses did not reveal significant differences across the frontal electrode cluster for the LPP; however, an exploratory single electrode analyses identified a significant effect at F1. Given the absence of an effect in the primary cluster-level analysis, this localised effect should be interpreted cautiously. Although it may suggest cognitive processing in response to the threat stimulus, further investigation is required to establish any functional significance. Together, these findings provide insight into the behavioural differences underlying the threat imminence continuum and defence responses in a virtual reality environment, whereas evidence for neural mechanisms remains exploratory.

## Data Availability

The raw data supporting the conclusions of this article will be made available by the authors upon request, without undue reservation.
