# Supplementary material for: Investigation of human defence responses in a virtual reality environment: an EEG study
Source: Front Hum Neurosci. 2026 Sep 30;20:1924058. doi: 10.3389/fnhum.2026.1924058 (PMC13661590; doi:10.3389/fnhum.2026.1924058)
Supplement: Supplementary file 1 [file Supplementary_file_1.docx]

**Supplementary File**

**References for preliminary study and main experiment**

- Punching and running animation and threat avatar (Adventure Blake: ManNeko, 2019, from the Unity Asset Store). License agreement: Standard Unity Asset Store EULA. License type: Extension Asset. <https://assetstore.unity.com/packages/3d/characters/humanoids/adventurer-blake-158728>
- Assets for the Environment (CITY package: 255 pixel studios, 2018, from Unity Asset Store). License agreement: Standard Unity Asset Store EULA. License type: Extension Asset. <https://assetstore.unity.com/packages/3d/environments/urban/city-package-107224>
- Auditory stimuli of the participant's footsteps during movement: footsteps shoes walk road asphalt hard.flac by kyles -- https://freesound.org/s/637556/ -- License: Creative Commons 0
- Running of the threat avatar: Running footsteps on pavement.WAV by thomasanthony321 -- https://freesound.org/s/682771/ -- License: Creative Commons 0

**Audio Sounds for preliminary study:**

- Audio_1: scream_01.flac by cdrk -- https://freesound.org/s/21323/ -- License: Attribution 4.0
- Audio_2: scream_01.flac by cdrk -- https://freesound.org/s/21323/ -- License: Attribution 4.0
- Audio_3: scottstoked_thinkfunny.wav by shawshank73 -- https://freesound.org/s/123748/ -- License: Attribution 3.0
- Audio_4: Male voice screaming loudly by Dinsfire -- https://freesound.org/s/169628/ -- License: Creative Commons 0
- Audio_5: Screams of frustration 2 by jorickhoofd -- https://freesound.org/s/180342/ -- License: Attribution 4.0

**Preliminary Study**

**Figure S1**

*Auditory Valence Ratings*

Auditory Valence Ratings

Valence Ratings (1=Unpleasant to 9=Pleasant)

| Auditory Stimuli | Valence_Mean |
| --- | --- |
| Audio_1 | 2.72727 |
| Audio_2 | 3.54545 |
| Audio_3 | 3.90909 |
| Audio_4 | 2.54545 |
| Audio_5 | 2.09091 |

**Figure S2**

*Auditory Arousal Ratings*

Auditory Arousal Ratings

Arousal Ratings (1=Calm to 9=Excited)

| Auditory Stimuli | Arousal_Mean |
| --- | --- |
| Audio_1 | 5.45455 |
| Audio_2 | 5.63636 |
| Audio_3 | 5.72727 |
| Audio_4 | 6 |
| Audio_5 | 5.81818 |

**Figure S3**

*Graph depicting perceived distance of the threat avatar*

Perceived Distance of the Threat Avatar

Ratings(1=Distal, 2=Not Sure, 3=Proximal)

| Perceived Distance of the Threat Avatar | Mean |
| --- | --- |
| Distal Threat Escape | 1.36364 |
| Proximal Threat Escape | 3 |
| Distal Threat No Escape | 1.36364 |
| Proximal Threat No Escape | 2.54545 |

**Figure S4**

*Graph showing the walking speed of the participant avatar*

Walking Speed of the Participant Avatar

Speed Ratings (1=Too Slow to 5=Too Fast)

| Trials | Speed_Mean |
| --- | --- |
| Familiarisation Scene | 2.27273 |
| No Threat | 2.72727 |

**EEG single electrode ERP grand average graphs**

**Figure S5**

*Grand average ERP graph for F4 electrode*

**Figure S6**

*Grand average ERP graph for F2 electrode*

**Figure S7**

*Grand average ERP graph for Fz electrode*

**Figure S8**

*Grand average ERP graph for F3 electrode*

**Figure S9**

*Grand average ERP graph for Fpz electrode*

**Figure S10**

*Grand average ERP graph for Fp2 electrode*

**Figure S11**

*Grand average ERP graph for Fp1 electrode*

**Preliminary Study Questionnaire for Virtual Reality Scenes**

| **Name:** | **Participant ID:** |
| --- | --- |
| **Age:** | **Gender:** |
| **Signature:** | **Date:** |

**INSTRUCTIONS**

In this experiment, you will be viewing a series of scenes within a 3D virtual environment on a desktop screen. In the 3D environment, you will be provided with instructions on how to navigate the environment and your task in that scene. After the end of each scene, a prompt will appear on the screen. Please take a moment to reflect on your experience during the scene and indicate your choice on this paper. Remember, there are no right or wrong answers; we are interested in your perception of the scene.

Your responses are valuable and will contribute significantly to the research.

**Read the statements below and mark the appropriate choice.**

**Scene 1:**

a) **Indicate your walking speed (of your character) in the virtual environment.**

**[	]** Too slow	**[	]** Slow	**[	]** Just right	**[	]** Fast	**[	]** Too fast

**Scene 2:**

a) **How did the enemy threat avatar first appear in terms of distance when it started approaching?**

**[	]** Distal (Far)	**[	]** Not Sure	**[	]** Proximal (Near/Close)

**Scene 3:**

a) **How did the enemy threat avatar first appear in terms of distance when it started approaching?**

**[	]** Distal (Far)	**[	]** Not Sure	**[	]** Proximal (Near/Close)

**Scene 4:**

a) **Indicate your walking speed (of your character) in the virtual environment.**

**[	]** Too slow	**[	]** Slow	**[	]** Just right	**[	]** Fast	**[	]** Too fast

**Scene 5:**

a) **How did the enemy threat avatar first appear in terms of distance when it started approaching?**

**[	]** Distal (Far)	**[	]** Not Sure	**[	]** Proximal (Near/Close)

**Scene 6:**

a) **How did the enemy threat avatar first appear in terms of distance when it started approaching?**

**[	]** Distal (Far)	**[	]** Not Sure	**[	]** Proximal (Near/Close)

**Additional Feedback:**

**Pre-Experiment Questionnaire**

**Santa Barbara Sense-of-direction scale**

The following statements ask you about your spatial and navigational abilities, preferences, and experiences.

1. I am very good at giving directions.

2. I have a poor memory for where I left things.

3. I am very good at judging distances.

4. My “sense of direction” is very good.

5. I tend to think of my environment in terms of cardinal directions (N, S, E, W).

6. I very easily get lost in a new city.

7. I enjoy reading maps.

8. I have trouble understanding directions.

9. I am very good at reading maps.

10. I don’t remember routes very well while riding as a passenger in a car.

11. I don’t enjoy giving directions.

12. It’s not important to me to know where I am.

13. I usually let someone else do the navigational planning for long trips.

14. I can usually remember a new route after I have traveled it only once.

15. I don’t have a very good “mental map” of my environment.

*Rating scale: 1=Strongly agree to 7=Strongly disagree*

**Post-Experiment Questionnaire**

**Instructions**

Thank you for participating in our Virtual Reality (VR) experiment. Your feedback is crucial in helping us understand the user experience in virtual environments. Please take a moment to complete the following questionnaire. Your responses will remain confidential and be used solely for research purposes.

Reflect on your experience during the VR experiment. Read each question carefully and provide your response based on your experience. For questions with rating scales, select the option that best represents your experience.

**Statements**

**Body-illusion Questionnaire** (Penaud et al., 2023).

**Sometimes during navigation…**

1. **I felt I had control over the body in the VR environment.** (Agency)
2. **I felt that the body in the VR environment was my own body.** (Self-identification)
3. **I had the feeling that I was located in the same place as the body in the VR environment.** (Self-location)
4. **I had the feeling that I couldn’t control my body anymore.** (Control)
5. **I felt I had two bodies.** (Control)

*7-point Likert Rating scale (-3=Strongly Disagree, 0=Neutral, 3=Strongly Agree).*

**i-group presence questionnaire (IPQ)** (modified to include only relevant items; Schubert et al., 2001)

1. In the computer generated world I had a sense of "being there". (not at all--very much)
2. Somehow I felt that the virtual world surrounded me. (fully disagree—fully agree)
3. I felt like I was just perceiving pictures. (fully disagree—fully agree)
4. I did not feel present in the virtual space. (did not feel—felt present)
5. I had a sense of acting in the virtual space, rather than operating something from outside. (fully disagree—fully agree)
6. How aware were you of the real world surrounding while navigating in the virtual world? (i.e. sounds, room temperature, other people, etc.)? (extremely aware-moderately aware-not aware at all)
7. How real did the virtual world seem to you? (completely real--not real at all)
8. How much did your experience in the virtual environment seem consistent with your real-world experience? (not consistent-moderately consistent-very consistent)
9. How real did the virtual world seem to you? (about as real as an imagined world--indistinguishable from the real world)

*(-3 to +3. Rating labels vary according to the question items)*

**A User Experience Questionnaire for VR Locomotion: Formulation and Preliminary Evaluation** (modified to include only relevant items; Boletsis, 2020).

1. I was interested in the VR experiment. (Immersion)
2. I felt boredom during the VR experiment. (Negative Affect)
3. I felt frustrated during the VR experiment. (Tension)
4. I had to put a lot of effort into understanding and navigating the VR environment. (Challenge)
5. I felt exhausted during the VR experiment. (Tiredness)
6. I felt very confident navigating the VR environment. (Perceived usability)

*A five-point Likert scale of 0 (not at all), 1 (slightly), 2 (moderately), 3 (fairly), and 4 (extremely).*

**Subjective dread ratings** (Modified; Lu et al., 2023)

1. How would you rate your level of anxiety in response to the situation depicted in the picture? (Safety)

2. How would you rate your level of anxiety in response to the situation depicted in the picture? (Pre-encounter)

3. How would you rate your level of anxiety in response to the situation depicted in the picture? (Distal threat)

4. How would you rate your level of anxiety in response to the situation depicted in the picture? (Proximal threat)

*0= (no dread) to 9= (maximum dread).*

**Virtual reality environment questionnaire** (Adapted and modified from Lu et al., 2023)

1. To what extent have you experienced the situation as if it was real? (Realism)
2. Did you feel at any time that you were vulnerable? (Vulnerable)
3. How violent do you find the scenes were? (Violent)
4. Did you think that you could be physically assaulted during the scene? (Assaulted)
5. How fearful did you feel when facing the human avatar? (Fear of threat avatar)
6. The sense of uncomfortable and fear increased when the avatar got closer to me. (Fear of approaching avatar)
7. How fearful did you feel when the human avatar first appeared in proximal (close) location? (Proximal threat)
8. How fearful did you feel when the human avatar first appeared at a distal (far) location? (Distal Threat)
9. How fearful did you feel when the human avatar appeared from the end of the lane and you were at the start of the lane? (Possibility of Escape)
10. How fearful did you feel when the human avatar appeared from the start of the lane and you were at the end of the lane? (No escape)

*Ratings on a 7-point scale (1=not at all, 4=neutral, 7=completely).*

**Table S1**

*Post-experiment Virtual Reality Questionnaires (Mean and SD)*
